# A systematic environmental intervention, nidotherapy, given to whole communities: protocol for a randomised stepped-wedge trial

**DOI:** 10.1192/bjo.2024.861

**Published:** 2025-04-11

**Authors:** Peter Tyrer, Mike Crawford, Abdullah Ahmad, Barbara Barrett, Woody Caan, Conor Duggan, Eleni Frisira, Tim Kendall, Jacob King, David Daley, Elizabeth Mullins, Richard Parish, Yangang Xing, Min Yang

**Affiliations:** Division of Psychiatry, Imperial College, London, UK; School of Social Sciences, Nottingham Trent University, Nottingham, UK; Department of Gerontology, Nottingham University Hospitals NHS Trust, Nottingham, UK; King’s Health Economics, London, UK; Royal Society of Public Health, London, UK; University of Nottingham, Nottingham, UK; Institute of Mental Health, School of Medicine, Mental Health and Clinical Neurosciences, University of Nottingham, Nottingham, UK; Nottinghamshire Healthcare NHS Foundation Trust, Nottingham, UK; University of Sheffield, Sheffield, UK; National Collaborating Centre for Mental Health, London, UK; School of Medicine, University of Nottingham, Nottingham, UK; Department of Public Health, University of Chester, Chester, UK; Department of Architecture, Design and the Built Environment, Nottingham Trent University, Nottingham, UK; Faculty of Arts and Design, Swinburne University of Technology, Melbourne, Australia; School of Public Health, Sichuan University, Chengdu, China

**Keywords:** Community interventions, nidotherapy, personality, social function, stepped-wedge cluster-randomised trial

## Abstract

**Background:**

Environmental changes can be positive in mental illness. Systematic, planned and guided environmental change in all its aspects is called nidotherapy. It has shown some benefit but has not been extended to whole communities.

**Aims:**

A cluster-randomised step-wedge trial is planned in six village communities in Nottinghamshire, England, covering an adult population of 400.

**Method:**

Adults in six villages will be offered a full personal environmental assessment followed by agreed change in different 3-month periods over the course of 1 year. All six villages have populations between 51 and 100 residents and are similar demographically. Assessments of mental health, personality status, social function, quality of life and environment satisfaction will be made. After the initial baseline period of 3 months, two villages will be randomised to nidotherapy for 3 months, a further two at 6 months and the last two at 9 months.

**Results:**

The primary outcome will be change in social function; secondary outcomes include health-related quality of life, anxiety and depressive symptoms, personality status, costs of nidotherapy and life satisfaction. Adverse events will also be recorded. The analysis will be carried out using a multimodal statistical approach examining (a) the change in scores of the primary outcome (social function); (b) change in scores of all secondary outcomes, including costs; and (c) changes in environmental satisfaction.

**Conclusions:**

The findings of this study should help to determine whether nidotherapy has a place in the early detection and treatment of mental pathology.

There has been considerable interest in the positive effects of environmental change in the management of mental illness and the promotion of well-being. Many of these have involved exposing people to natural surroundings, particularly the green environment, and there is growing evidence that such interventions improve mental health mainly by reducing depression, anxiety and stress-related symptoms.^1–5^ These interventions are not usually introduced for specific health problems in the mentally ill apart from lifestyle elements to improve cardiovascular health.^2^ Community studies have also shown that greening unsatisfactory waste environments has a positive impact on the mental functioning of communities.^6–9^

However, all of these interventions are decided by external agencies, not by people or patients themselves. Nidotherapy, a planned personal collaborative environmental change, was developed in 2002^10^ and has been in use in the National Health Service (NHS) and internationally for 25 years but only in a few limited places. Nidotherapy is an individual therapy but differs from all other psychotherapies in that it focuses entirely on changing the environment, not the person, and has been highly praised for its attention to individual needs.^11^ Although it is superficially similar to other environmental interventions such as social prescribing, it differs in that patients themselves choose the environmental interventions they would like to have implemented and the therapist assesses these systematically with good knowledge of an individual’s strengths and weaknesses. The task of the nidotherapist is to evaluate proposed interventions against feasibility, and then help to facilitate their attainment.^12^

Nidotherapy has been tested in two small controlled trials, one in people with severe mental illness and the other in people with intellectual disability. The first of these was carried out in patients with severe mental illness and personality disorder in an inner-city service. This showed evidence of cost-effectiveness by reducing hospital in-patient care and promoting better community placement,^13^ improving social function in those with comorbid substance use^14^ and meriting a Cochrane review.^15^ The second trial was carried out in patients with intellectual disability who showed challenging behaviour and, in view of the limited mental capacity of the patients, the training in nidotherapy was carried out with staff. This trial also showed benefit in reducing challenging behaviour compared with the enhanced care programme approach, but the benefits did not appear immediately.^16^ There has also been another study showing problems in providing choice in nidotherapy in forensic patients,^17^ a series of case studies showing long-term benefit after treatment^18,19^ and also the use of nidotherapy in drama.^20,21^

The proposed study intends to extend the scope of nidotherapy into a whole population setting where it is recognised that there will be much less serious mental pathology but more opportunity for individual needs to be matched up with those of others in the community. For example, one of the main causes of poor mood in elderly people living alone is loneliness. Interventions to improve this have had limited success^22^ but in a community setting environmental interventions to improve social involvement could be made easily if fellow neighbours were able to meet other lonely individuals in common activities. These could be facilitated by nidotherapy.

## Research objectives

The primary objective of the trial is to determine if nidotherapy given in a whole community setting is more effective in improving social function than a mere demonstration of nidotherapy principles in other whole communities. The secondary objectives are to determine if nidotherapy also improves depressive and anxiety symptoms, quality of life, personality functioning and satisfaction with care to a greater extent than just demonstration of principles. We also wish to determine if nidotherapy is cost-effective by recording all the costs associated with its administration.

## Method

The trial design is a cluster-randomised controlled trial using a step-wedge design of nidotherapy to be given to all adult residents (18 or over) in six villages in Nottinghamshire in central England. The plan is to randomise the six villages into three clusters each containing two villages, with active nidotherapy being given to each cluster for a period of 3 months. As the project will explain the principles of nidotherapy from the start of the study, when the people in villages are not receiving active nidotherapy, they will represent an unexposed passive nidotherapy control population.

### Study setting

The six villages are Cotham, Hawton, Kilvington, Alverton, Thorpe and Staunton (total estimated eligible population, 300). Each village has similar population demographic characteristics with most residents over the age of 60, with no economically deprived areas, with most people living in owned properties. Each village has a church but no shops or other amenities.

### Eligibility criteria

The intention is to recruit all who satisfy the eligibility criteria to be involved in the trial in some form.

#### Inclusion criteria

Aged 18 or over living in one of the six identified villages.

#### Exclusion criteria

Impaired mental capacity leading to inability to consent; serious physical illness preventing the possibility of planned environmental change, to be assessed by the clinician and external medical advice; inability to speak English sufficiently well to understand all parts of the trial.

### Recruitment

This will be achieved through village meetings organised by parish groups, leaflets, word-of-mouth snowballing, related events and church communications. Preliminary work has found that general meetings followed by word-of-mouth link-up has been the most productive pathway.

### Statistical design

The trial will use a randomised clustered step-wedge design with two arms. In the first 3-month period, the residents in all villages will be assessed to evaluate their clinical status and their environmental wishes. After 3 months a short check will be made on any material changes since recruitment. Two randomisations will then be carried out in three steps over 1 year. After the first 3 months of baseline assessment, two of the villages will be independently randomised to receive nidotherapy, with the other four acting as unexposed controls. At 6 months, another two villages from the remaining four will be randomised to nidotherapy with the last two unexposed villages as controls; at 9 months these remaining two villages will receive nidotherapy. Thus, the two villages receiving nidotherapy at first randomisation will have three 3-month periods to observe the post-treatment effects, the second two will have two periods to observe the post-treatment effects and the last two villages will have nidotherapy completed 12 months after the trial starts, and a final follow-up will take place for all participants within the following 3 months, and at 6 and 9 months environmental and short clinical assessments will be repeated on all participants.

The analysis will be carried out using intention-to-treat together with imputation of missing data. Analysis will be separated into three components: (a) change in scores of the primary outcome (social function); (b) change in scores of all secondary outcomes, including costs; and (c) changes in environmental satisfaction. Comparison in mean changes of outcomes between the two groups will be conducted using random effects models or multilevel models under a generalised linear modelling framework. Multilevel models^23^ will take into account both cluster effects of villages and difference among individuals, while estimating intervention effects over time. Considering that participants are not being randomised into treatment groups, refined models to assess intervention effects could adjust for baseline measures and other possible confounders.

### Interventions

#### All villages

The eligible inhabitants of all villages will receive an explanation of nidotherapy and a rounded assessment of their present circumstances, their personal strengths and motivations and mental health status. This is presented as a cursory mental health assessment but with no further intervention. A good assessment of general functioning and personality is an important part of environmental selection in nidotherapy.

#### Nidotherapy villages

The procedure described for all villages will be followed, but in the active nidotherapy villages further assessments before any changes will include an environmental analysis involving social, physical and personal aspects, matching of personality characteristics with the development of an environmental intervention using a formal procedure (or if no intervention is required, a plan for future change) and a timetable (nidopathway) with subsequent monitoring of progress.^24^ As the choice of environmental change is made by the patient, the intervention course cannot be predicted in advance; however, in most cases we expect that the main components will be completed within 2 months, and for the purposes of the trial all facilitated interventions will be completed by 3 months.

Nidotherapy will be administered by trained environmental facilitators under the supervision of P.T., who has completed training in the subject by a combination of theoretical learning and practice under supervision. This enables a full assessment of personality strengths and motivations and allows the right choice of intervention to follow. Some of the practical aspects of achieving environmental change may also require nidotherapy volunteers who have also been trained in the principles of nidotherapy. Each nidotherapy intervention will be classified by a central team as an individual intervention (one designed for that person or family alone) or a community intervention (defined as one that requires the support and involvement of at least 10 members of the village). In some cases, both individual and community interventions may satisfy these definitions.

Examples of the changes achieved in nidotherapy range from community ones to improve social isolation to employment interventions, improving local amenities, help in relationships and bigger changes such as a change of housing arrangements. The main advantage of community involvement is joint decision-making between members of the village, allowing shared benefits to be attained. If nidotherapy is embraced across the village, further benefits could be achieved. In many studies of desired interventions, equipoise is not present. In this study, because we have no previous data, equipoise is achieved, as it is quite possible that residents in the villages may be discomfited by assessments of their mental health and satisfaction, and view any attempts at intervention as interference in the smooth life of the village. The population being studied is not a typical one and is unlikely to be representative of the population with respect to mental health, even though suicide rates are higher in rural than urban areas. However, it has the advantage of being a relatively stable population whose circumstances are normally unchanging in the short term.

### Qualitative project

We plan to conduct qualitative work towards the end of the trial to achieve the following.

- Assess the acceptability and feasibility outcomes of nidotherapy.
- Identify the factors associated with changes in outcome reporting.

A random non-stratified sample of trial completers will be approached, and those who consent will undergo semi-structured individual interviews, mainly in-person or via teleconferencing, during or after the final follow-up period. An interview guide will be developed and will likely contain prompts on the following:

- participants’ initial expectations of the trial;
- their experience of the assessment;
- their reaction to the nidotherapy intervention plan;
- their views on the acceptability and feasibility of individual- or community-level interventions when relevant;
- the areas of positive change in themselves or their community that they link to the intervention;
- areas of less positive changes that they associate with the intervention.

These prompts have been chosen from clinical experience and the results of previous studies of nidotherapy. Interviews will be recorded and transcribed, with audio and text stored securely in line with General Data Protection principles. Data will be coded using Lumivero NVivo (version 15 for Mac OS; Lumivero, Denver, Colorado, USA; https://lumivero.com/products/nvivo/). A minimum of five participants will be selected, after which point participants will continue to be recruited until thematic saturation is reached (we anticipate this to be between 7 and 12 people – see https://www.ncbi.nlm.nih.gov/pmc/articles/PMC7200005/). A summarising description of limited non-identifiable demographic details (age, gender, brief description of the participant’s intervention) will be included for those interviewed. This qualitative work will follow the consolidated criteria for reporting qualitative research (COREQ) guidelines.

### Ethical aspects

The study has been approved by the Schools of Business, Law and Social Sciences Research Ethics Committee (BLSS REC) (project number 1900275) at Nottingham Trent University. All those taking part in the study will receive a participation information sheet and complete a signed consent form. We will follow the Helsinki Declaration criteria in that participants will have the right to cease involvement in the study and to withdraw from the trial at any time and for any reason, without prejudice to any future care. An investigator may also withdraw a participant from the trial at any time in the interests of the participant’s health and well-being or for administrative reasons.

To improve participation in the trial there will be open meetings at centres in each of the villages and invitation for all to take part in the merits of agreed environmental change.

### Assessments

#### Baseline

All participants will receive a baseline assessment to determine eligibility and agreement to take part in all parts of the trial. This will be preceded by publicity from local news outlets.

Each of the participants agreeing to take part will complete the following assessments, deliberately chosen to be relatively short and easy to complete in all groups:

- the Short Social Functioning Questionnaire (SSFQ),^25^ a short version of a well-tested measure;^26^
- the Structured Assessment of Personality – Abbreviated Scale (SAPAS);^27^
- the Assessment of Personality Strengths Scale (APSS);^28^
- the Hospital Anxiety and Depression Scale (HADS);^29^
- the Personality Assessment Questionnaire for ICD-11 – Revised (PAQ-11-R);^30^
- the Recovering Quality of Life (ReQoL) (10-item version);^31^
- the Personality Assessment Schedule for ICD-11 (PDS-ICD-11);^32^
- the PROMIS-Short Form for Social Isolation (PROMIS-SF) (a four-item scale for social cohesion) at initial and last assessment;^33^
- a satisfaction scale and the SSFQ^25^ will be completed at the last assessment.

These data will be help to create a combined assessment of personality, with the Structured Personality Assessment from Notes and Documents (SPAN-DOC)^34^ tool.

These assessments will be made at interview and or by self-completed paper records immediately stored at a data protected facility to ensure confidentiality. These data will represent a mental health profile of each resident at each stage of the study.

Details of all nidotherapy actions will be recorded to establish the total costs of interventions.

**Fig. 1:**
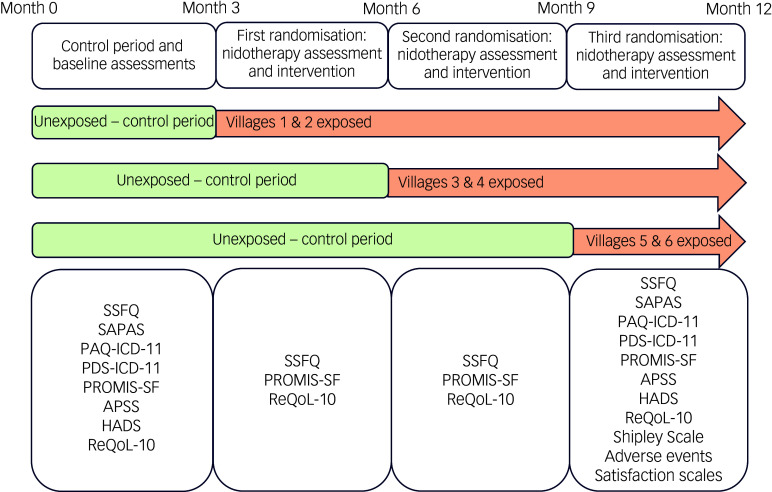
Project timeline. APSS, Assessment of Personality Strengths Scale; HADS, Hospital Anxiety and Depression Scale; PAQ-ICD-11-R, Personality Assessment Questionnaire for ICD-11 – Revised; PDS-ICD-11, Personality Assessment Schedule for ICD-11; PROMIS-SF, PROMIS-Short Form for Social Isolation; ReQoL, Recovering Quality of Life – 10 items; SAPAS, Structured Assessment of Personality – Abbreviated Scale; SSFQ, Short Social Functioning Questionnaire.

After 3 months of initial data collection a short check will be made on any material changes since early recruitment. Two of the villages will then be randomised to nidotherapy independently by M.Y. (Fig. 1).

### Second assessment: three months after nidotherapy input to the first two villages

The SSFQ, PROMIS and ReQoL assessments will be completed for all participants.

*Third assessment: 6 months after completion of baseline assessment and after nidotherapy input to the second two villages*

The SSFQ, PROMIS and ReQoL assessments will be completed for all participants.

*Fourth assessment: 9 months after completion of baseline assessment and after nidotherapy input to the last two villages.*

All the assessments at baseline will be repeated, together with satisfaction scales, including an adverse event scale derived from the work of Klatte et al^35^ to determine the nature of adverse events in either arm of the trial. Satisfaction with the village environment will be recorded as a general statement at all assessments and satisfaction with the interventions also assessed using an agreed scale.^36^

A record of the most positive and negative environmental events^37^ will be made at the termination of the trial and linked to the cost-effectiveness analysis.

## Discussion

This study represents one of the first interventions in preventive psychiatry. If communities are able to respond positively and appropriately to the needs of those who are liable to become mentally ill, they would serve a valuable public health service. We recognise that six rural villages are not in any way representative of the population at large, but they allow evaluation of the effects of intervention in a way that might be more difficult elsewhere.

## Data Availability

Relevant anonymised data from this study will be available from the statistician, M.Y., via the corresponding author, P.T.
